# Supplementary material for: Cycle-by-cycle respiration waveforms are coupled with the shape of neural oscillations
Source: bioRxiv. 2026 Apr 14:2026.04.13.718339. Preprint. [Version 1] doi: 10.64898/2026.04.13.718339 (PMC13104959; doi:10.64898/2026.04.13.718339)
Supplement: 1 [file NIHPP2026.04.13.718339v1-supplement-1.pdf]

bioRxiv preprint doi: https://doi.org/10.64898/2026.04.13.718339; this version posted April 14, 2026. The copyright holder for this preprint (which was not certified by peer review) is the author/funder, who has granted bioRxiv a license to display the preprint in perpetuity. It is made available under aCC-BY 4.0 International license.

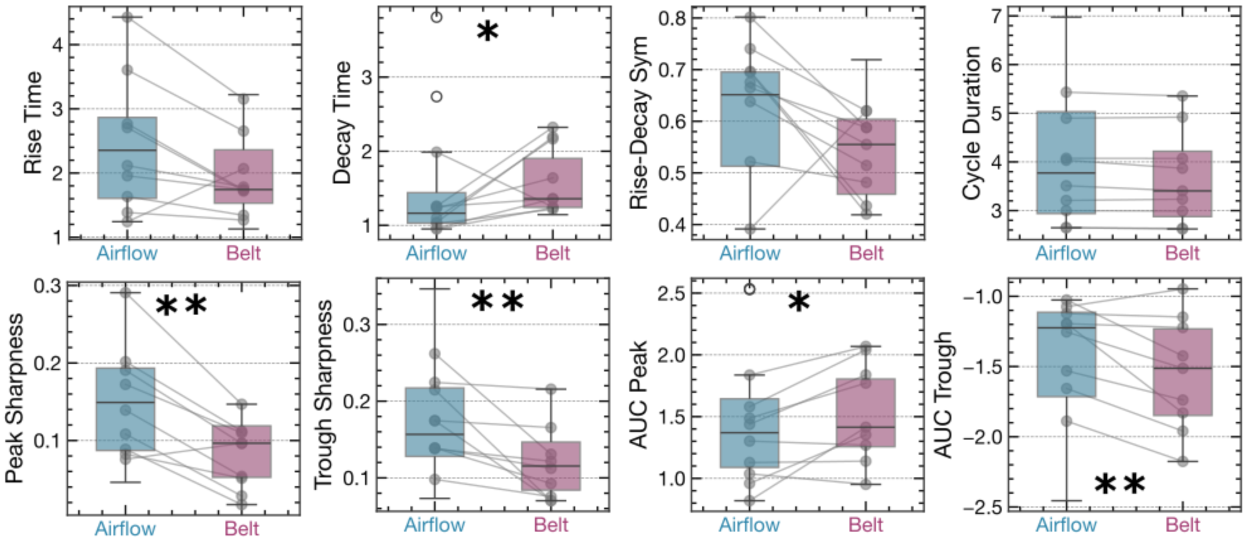

Fig. S1 | Respiration waveform shape quantified across modalities.
Boxplots show the distribution of median per-participant waveform shape features for airflow (blue) and belt (purple) recordings for participants with both modalities (N = 10). Each point represents one participant; gray lines connect paired observations from the same participant (n = 10 participants with both modalities). Asterisks indicate significant paired differences by Wilcoxon signed-rank test (* $p < 0.05$, ** $p < 0.01$).
